# Selective excitation of spin-polarized alkali atoms in different ground-state hyperfine levels

**DOI:** 10.1038/s41598-017-14431-8

**Published:** 2017-11-20

**Authors:** Zhichao Ding, Jie Yuan, Yangying Fu, Hui Luo, Xingwu Long

**Affiliations:** College of Optoelectronic Science and Engineering, National University of Defense Technology, Changsha, 410073 China

## Abstract

A technique to selectively excite spin-polarized alkali atoms in one of the two ground-state hyperfine levels is demonstrated, which can separately create the transverse spin component of spin-polarized alkali atoms in either ground-state hyperfine level. The principle of this technique is analyzed, and the experimental results are found to be in good agreement with the theoretical predictions, thereby demonstrating the feasibility of this technique. This technique can be used to accurately measure spin relaxation and polarization of alkali atoms in either ground-state hyperfine level. An example of its applications to measure the transverse relaxation time is presented.

## Introduction

**Figure 1:**
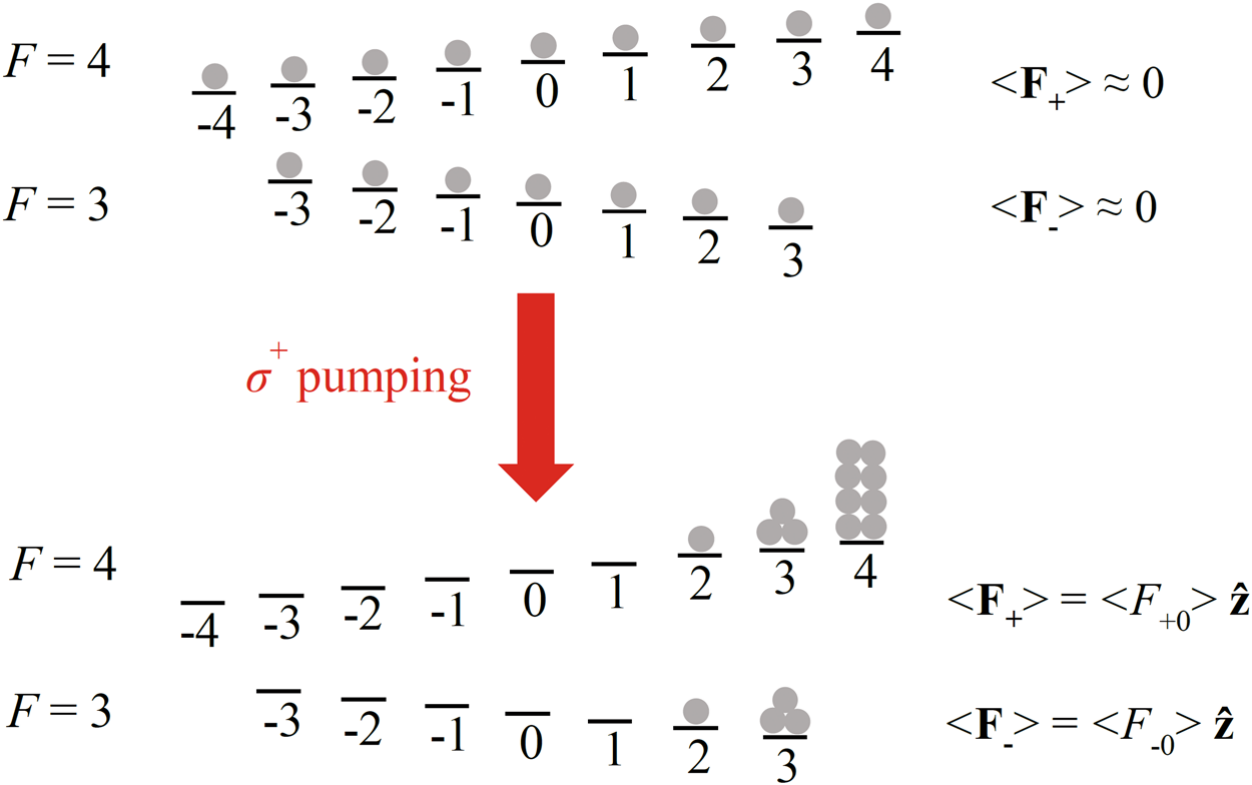
Optical pumping of a ^133^Cs ensemble with left circularly polarized light *σ* ^+^ in a static magnetic field along the z-axis. *F* = 3 and *F* = 4 respectively represent the lower and the upper ground-state hyperfine levels of a ^133^Cs atom, and their corresponding Zeeman sublevels are shown with the horizontal lines. The gray balls indicate the atomic populations. 〈**F**_−_〉 and 〈**F**_+_〉 are the expectation values of the total angular momentum of alkali atoms in the lower and the upper ground-state hyperfine levels, respectively. $\langle {F}_{+0}\rangle ={\sum }_{{m}_{F}=-4}^{4}{m}_{F}\rho (F=4,{m}_{F})\hslash$ and $\langle {F}_{-0}\rangle ={\sum }_{{m}_{F}=-3}^{3}{m}_{F}\rho (F=3,{m}_{F})\hslash$, where *m*_*F*_ is the magnetic quantum number, $\rho (F,{m}_{F})$ is the probability of a ^133^Cs atom being in Zeeman state |*F*, *m*_*F*_〉, and $\hslash$ is the reduced Plank constant.

For an ensemble of alkali atoms in the presence of a static magnetic field, the atomic populations of the ground-state Zeeman sublevels are almost the same in equilibrium, and the expectation value of the total angular momentum of alkali atoms is approximately equal to zero. When circularly polarized light propagating along the static magnetic field direction is applied to pump this ensemble as shown in Fig. 1, the photons add angular momentum to the alkali atoms, creating population difference of alkali atoms in different ground-state Zeeman sublevels and leading to the spin polarization of alkali atoms^1–3^. The spin-polarized atomic ensemble is a powerful medium, which is extensively employed in a variety of significant fields, such as atomic magnetometers^4–7^, nuclear magnetic resonance^8,9^, Faraday filters^10,11^, and quantum memory and teleportation^12,13^.

**Figure 2:**
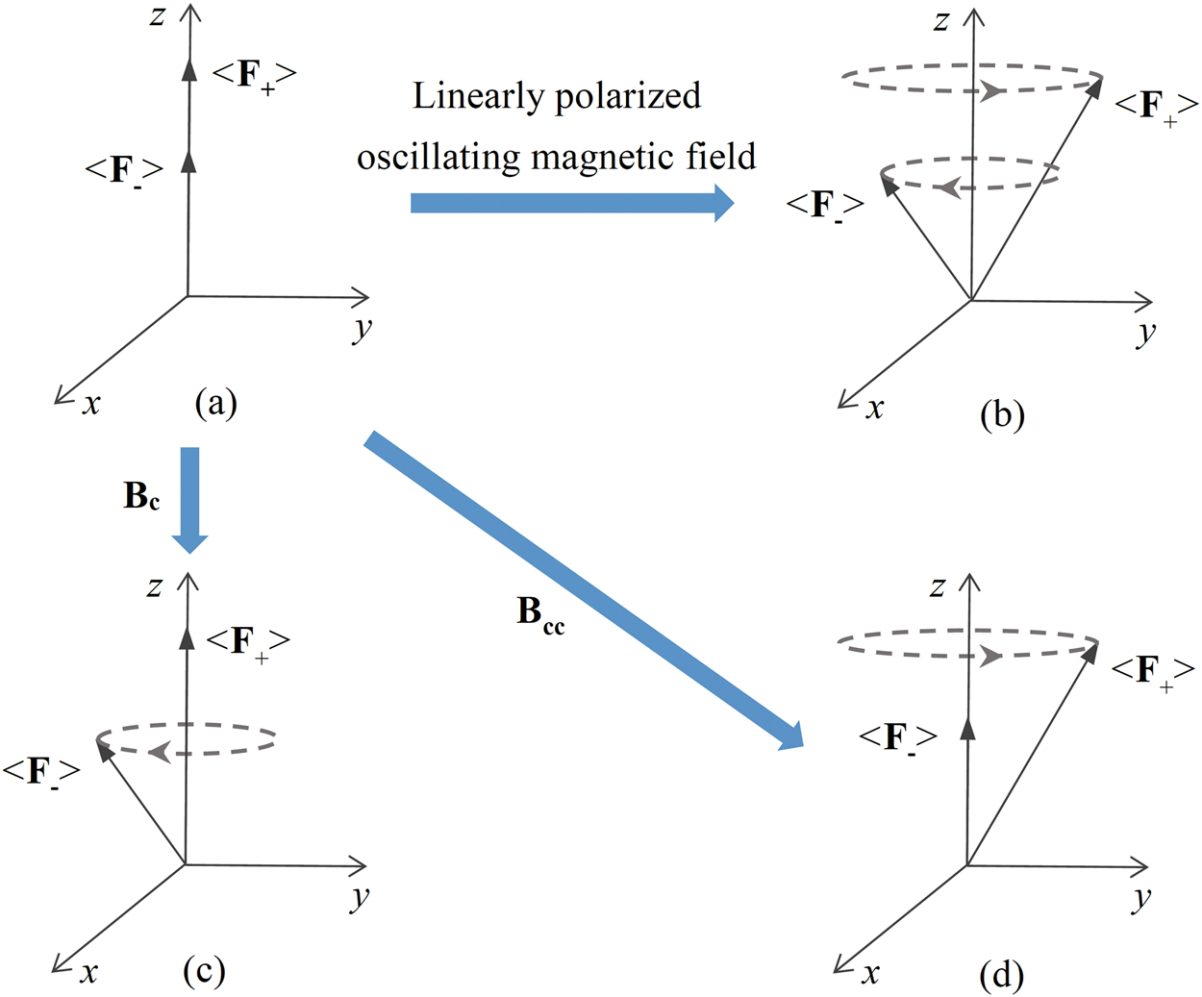
Excitation of optically pumped alkali atoms in a static magnetic field along the z-axis. (**a**) The initially spin-polarized state for optically pumped alkali atoms. (**b**), (**c**) and (**d**) the states when the optically pumped alkali atoms are excited by a linearly polarized oscillating magnetic field, a clockwise rotating magnetic field **B**_**c**_ and a counterclockwise rotating magnetic field **B**_**cc**_, respectively.

The spin polarization of optically pumped alkali atoms, which can be represented by the expectation value of the total angular momentum, is along the static magnetic field direction as shown in Fig. 1 and Fig. 2(a) ^1^. However, in many applications, the transverse spin component of a spin-polarized atomic ensemble, which is perpendicular to the static magnetic field, needs to be created. For example, by monitoring the transverse spin component, we can obtain some crucial parameters of the atomic ensemble, like the transverse relaxation time of atomic spins^14,15^. In addition, the transverse spin component is also used to extract information of external magnetic fields for a common type of atomic magnetometer, M_x_ magnetometer^16,17^.

The transverse spin component of spin-polarized alkali atoms is conventionally created by applying a linearly polarized oscillating magnetic field or employing modulated light^18,19^. Under the excitation of the linearly polarized oscillating magnetic field or modulated light, the atomic spins created by optical pumping will tilt away from and precess about the static magnetic field direction, thereby creating the transverse spin component. When the hyperfine structure is considered, the oscillating magnetic field or modulated light will excite magnetic dipole transitions and create transverse spin components of alkali atoms in both ground-state hyperfine levels as shown in Fig. 2(b). However, in many cases, we only want to excite alkali atoms in one of hyperfine levels especially when the hyperfine structure is resolved, such as a precise measurement of the transverse relaxation time of alkali atoms in a certain hyperfine level.

In this paper, we report a technique to selectively excite spin-polarized alkali atoms in different ground-state hyperfine levels. By controlling the direction of an applied rotating magnetic field, we can choose to excite magnetic dipole transitions and create the transverse spin component of alkali atoms in either hyperfine level as shown in Fig. 2(c) and (d). This technique can be used to accurately measure spin relaxation and polarization of alkali atoms in either ground-state hyperfine level, which is helpful for the research of optical pumping and relaxation mechanisms. In addition, this technique can also be applied into the M_x_ magnetometer to improve the measuring precision of external magnetic fields.

## Methods

### Theoretical Background

For an alkali ensemble in an ambient static magnetic field ${{\bf{B}}}_{{\bf{0}}}={B}_{0}\hat{{\bf{z}}}$, where $\hat{{\bf{z}}}$ is the unit vector along the z-axis, and ${B}_{0} > 0$ is the amplitude of the static magnetic field. While left circularly polarized light propagating along the z-axis is applied, the alkali ensemble will be polarized. From a macroscopic view, nonzero expectation values $\langle {{\bf{F}}}_{-}\rangle =\langle {F}_{-0}\rangle \hat{{\bf{z}}}$ and $\langle {{\bf{F}}}_{{\boldsymbol{+}}}\rangle =\langle {F}_{+0}\rangle \hat{{\bf{z}}}$ of the total angular momentum of alkali atoms in the lower ($F=I-1/2$) and upper ($F=I+1/2$) ground-state hyperfine levels are produced by optical pumping as shown in Figs 1 and 2(a). Here, *F* and *I* represent quantum numbers for the total atomic angular momentum and nuclear spin, respectively.

When a rotating magnetic field ${{\bf{B}}}_{{\bf{e}}{\bf{x}}}={B}_{1}\,\cos (\omega t)\hat{{\bf{x}}}-{B}_{1}\,\sin (\omega t)\hat{{\bf{y}}}$ is applied to excite this atomic ensemble, considering the alkali atoms in one of the two ground-state hyperfine levels, synthesizing the effects of optical pumping and spin relaxation which depolarizes atomic spins due to some spin-relaxation mechanisms^1^, the evolution of atomic spins represented by the expectation value 〈**F**〉 of the total angular momentum satisfies the following Bloch equation^16,20^:

1 $$\frac{{\rm{d}}\langle {\bf{F}}\rangle }{{\rm{d}}t}=\gamma \langle {\bf{F}}\rangle \times {\bf{B}}-\frac{\langle {F}_{x}\rangle \hat{{\bf{x}}}+\langle {F}_{y}\rangle \hat{{\bf{y}}}}{{T}_{2}}+\frac{\langle {F}_{0}\rangle -\langle {F}_{z}\rangle }{{T}_{1}}\hat{{\bf{z}}}.$$

Here, 〈**F**〉 = 〈**F**_−_〉 and 〈**F**〉 = 〈**F**_+_〉 for the alkali atoms in the lower and the upper ground-state hyperfine levels, respectively, and 〈**F**〉 is represented as $(\langle {F}_{x}\rangle ,\langle {F}_{y}\rangle ,\langle {F}_{z}\rangle )$ in the reference frame xyz. *γ* is the gyromagnetic ratio of alkali atoms in this ground-state hyperfine level. ${\bf{B}}={{\bf{B}}}_{{\bf{0}}}+{{\bf{B}}}_{{\bf{e}}{\bf{x}}}$ is the external magnetic field. $\hat{{\bf{x}}}$ and $\hat{{\bf{y}}}$ are the unit vectors along the x and y axes, respectively. ${T}_{2}$ and ${T}_{1}$ are respectively the transverse and the longitudinal relaxation times of atomic spins for this ground-state hyperfine splitting. 〈*F*_0_〉 = 〈*F*_−0_〉 and 〈*F*_0_〉 = 〈*F*_+0_〉 for the alkali atoms in the lower and the upper ground-state hyperfine levels, respectively.

In order to simplify the analysis, a rotating frame x′y′z which rotates around the z-axis with a frequency of $\omega$ is introduced. In the rotating frame x′y′z, $\langle {\bf{F}}\rangle$ is represented as $(\langle {F}_{x\text{'}}\rangle ,\langle {F}_{y\text{'}}\rangle ,\langle {F}_{z}\rangle )$ and given by

2 $$\{\begin{array}{c}\langle {F}_{x}\rangle =\langle {F}_{x\text{'}}\rangle \cos (\omega t)+\langle {F}_{y\text{'}}\rangle \sin (\omega t)\\ \langle {F}_{y}\rangle =\langle {F}_{y\text{'}}\rangle \cos (\omega t)-\langle {F}_{x\text{'}}\rangle \sin (\omega t)\end{array}.$$

Substituting equation (2) into equation (1) and setting ${\rm{d}}\langle {F}_{x\text{'}}\rangle /{\rm{d}}t={\rm{d}}\langle {F}_{y\text{'}}\rangle /{\rm{d}}t={\rm{d}}\langle {F}_{z}\rangle /{\rm{d}}t=0$, one can easily obtain the stationary solutions as follows

3 $$\{\begin{array}{c}\langle {F}_{x\text{'}}\rangle =\langle {F}_{0}\rangle \frac{-{\omega }_{1}{T}_{2}^{2}(\omega -{\omega }_{0})}{1+{\omega }_{1}^{2}{T}_{1}{T}_{2}+{T}_{2}^{2}{(\omega -{\omega }_{0})}^{2}}\\ \langle {F}_{y\text{'}}\rangle =\langle {F}_{0}\rangle \frac{{\omega }_{1}{T}_{2}}{1+{\omega }_{1}^{2}{T}_{1}{T}_{2}+{T}_{2}^{2}{(\omega -{\omega }_{0})}^{2}}\\ \langle {F}_{z}\rangle =\langle {F}_{0}\rangle \frac{1+{T}_{2}^{2}{(\omega -{\omega }_{0})}^{2}}{1+{\omega }_{1}^{2}{T}_{1}{T}_{2}+{T}_{2}^{2}{(\omega -{\omega }_{0})}^{2}}\end{array}.$$

Here, ${\omega }_{0}=\gamma {B}_{0}$ and ${\omega }_{1}=\gamma {B}_{1}$. By applying proper ${{\bf{B}}}_{{\bf{0}}}$ and ${{\bf{B}}}_{{\bf{e}}{\bf{x}}}$, the conditions that $|{\omega }_{1}|$≪$|{\omega }_{0}|$, ${T}_{1}$≫$1/|{\omega }_{0}|$, and ${T}_{2}\gg$ $1/|{\omega }_{0}|$ for the technique to selectively excite spin-polarized alkali atoms can be easily satisfied, which are also the common cases in practical applications^18^.

For the alkali atoms in the lower ground-state hyperfine level, the gyromagnetic ratio $\gamma ={\gamma }_{-} > 0$, while $\gamma ={\gamma }_{+}\approx -{\gamma }_{-} < 0$ for the alkali atoms in the upper ground-state hyperfine level^16,21^. According to equation (3), when the rotating magnetic field is clockwise with respect to **B**_**0**_ and $\omega \approx |{\omega }_{0}|$, $\langle {F}_{x\text{'}}\rangle \approx \langle {F}_{y\text{'}}\rangle \approx 0$ for the alkali atoms in the upper ground-state hyperfine level, while $\langle {F}_{y\text{'}}\rangle$ can be a large value for the alkali atoms in the lower ground-state hyperfine level. However, when the rotating magnetic field is counterclockwise with respect to **B**_**0**_ and $\omega \approx -|{\omega }_{0}|$, the results are just the reverse. Therefore, as shown in Fig. 2(c) and (d), we can apply a clockwise rotating magnetic field to just excite spin-polarized alkali atoms in the lower ground-state hyperfine level, and a counterclockwise rotating magnetic field to just excite spin-polarized alkali atoms in the upper ground-state hyperfine level.

### Experimental Implementation

In order to verify the above theoretical analysis, we need to measure the transverse spin components (x-components 〈*F*_−*x*_〉 and 〈*F*_+*x*_〉 or y-components) of spin-polarized alkali atoms in the two ground-state hyperfine levels under different exciting magnetic fields. Since the polarization plane of linearly polarized probe light propagating along the x-axis will rotate after passing through a vapor of spin-polarized alkali atoms, and the rotating angle *θ* is given by^21,22^

4 $$\theta ={k}_{-}\langle {F}_{-x}\rangle +{k}_{+}\langle {F}_{+x}\rangle ,$$

where *k*_−_ and *k*_+_ are related to the alkali vapor density and the probe-light frequency, we can use a linearly polarized light propagating along the x-axis to detect 〈*F*_−*x*_〉 and 〈*F*_+*x*_〉.

**Figure 3:**
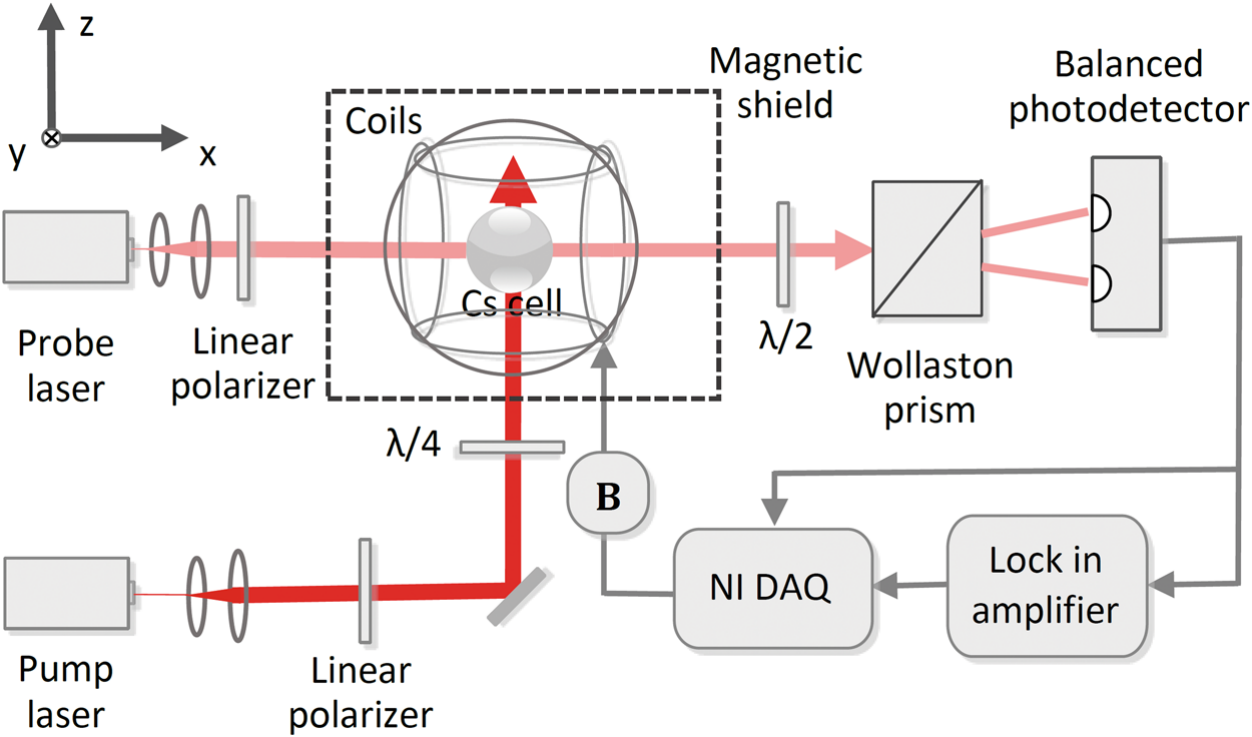
Schematic diagram of the experimental setup.

The experimental setup is shown in Fig. 3. A spherical Pyrex cell with a diameter of 20 mm is placed inside a five-layer μ-metal magnetic shield. The cell contains ^133^Cs atoms and buffer gas (50 Torr of N_2_ and 50 Torr of ^4^He) for slowing atomic diffusion and quenching. It is heated using two pairs of heating resistors driven by a high-frequency oscillating current with a frequency far away from the magnetic resonance frequency of ^133^Cs atoms, and its temperature is stabilized at 60 °C. Three pairs of Helmholtz coils, which are driven by a NI data acquisition system (DAQ), are used to generate the magnetic field experienced by ^133^Cs atoms. The static magnetic field **B**_**0**_ with ${B}_{0}=18.6\,\mu {\rm{T}}$ is generated by the pair of Helmholtz coils along the z-axis, and the exciting magnetic field **B**_**ex**_ with ${B}_{1}=2.9\,{\rm{nT}}$ is generated by the two pairs of Helmholtz coils along the x and y axes.

The pump and probe beams are generated by two distributed feedback diode lasers, which are detuned approximately 3.5 GHz towards lower frequency from the $F=3\to F^{\prime} =3$ component of the ^133^Cs D1 line. Their intensities are 100 μW/cm^2^ and 30 μW/cm^2^ and their beam sizes are 0.79 cm^2^ and 0.28 cm^2^, respectively. The pump beam becomes left circularly polarized light after passing through a linear polarizer and a λ/4 plate. Then, it polarizes the ^133^Cs atoms along the z-axis. After passing through a linear polarizer, the probe beam polarizes at an angle of 45° to the z-axis in the yz plane. Then, it illuminates the cell, and its polarization plane is detected using a setup involving a λ/2 plate, a Wollaston prism and a balanced photodetector. The λ/2 plate is adjusted to zero the output of the photodetector when the pump beam and magnetic field are not applied. The detected signal of the balanced photodetector, which is proportional to *θ* ^21^, is demodulated by a lock-in amplifier with the reference frequency of $\omega$, and the demodulated in-phase and quadrature signals are acquired by the NI DAQ. According to equations (2–4), the demodulated in-phase and quadrature signals are respectively proportional to ${k}_{-}\langle {F}_{-x\text{'}}\rangle +{k}_{+}\langle {F}_{+x\text{'}}\rangle$ and ${k}_{-}\langle {F}_{-y\text{'}}\rangle -{k}_{+}\langle {F}_{+y\text{'}}\rangle$. Meanwhile, the detected signal of the balanced photodetector is also directly acquired by the NI DAQ.

## Results and Discussion

**Figure 4:**
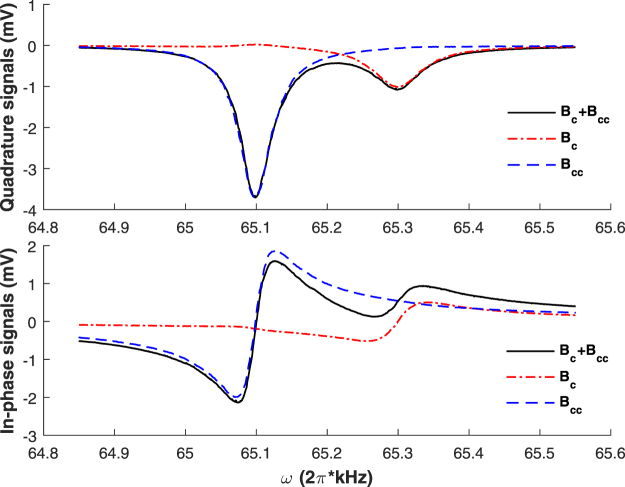
Magnetic resonance spectrums under different exciting magnetic fields: a clockwise rotating magnetic field **B**_**c**_, a counterclockwise rotating magnetic field **B**_**cc**_, and a linearly polarized oscillating magnetic field **B**_**c**_ + **B**_**cc**_.

Figure 4 shows the in-phase and quadrature signals of magnetic resonance spectrums when the frequency of the exciting magnetic fields is scanned. The red dash-dot lines, the blue dash lines, and the black solid lines are the experimental results when the exciting magnetic field is a clockwise rotating magnetic field ${{\bf{B}}}_{{\bf{e}}{\bf{x}}}={{\bf{B}}}_{{\bf{c}}}={B}_{1}\,\cos (\omega t)\hat{{\bf{x}}}-{B}_{1}\,\sin (\omega t)\hat{{\bf{y}}}$, a counterclockwise rotating magnetic field ${{\bf{B}}}_{{\bf{e}}{\bf{x}}}={{\bf{B}}}_{{\bf{c}}{\bf{c}}}={B}_{1}\,\cos (\omega t)\hat{{\bf{x}}}+{B}_{1}\,\sin (\omega t)\hat{{\bf{y}}}$, and a linearly polarized oscillating magnetic field ${{\bf{B}}}_{{\bf{e}}{\bf{x}}}={{\bf{B}}}_{{\bf{c}}}+{{\bf{B}}}_{{\bf{c}}{\bf{c}}}=2{B}_{1}\,\cos (\omega t)\hat{{\bf{x}}}$, respectively, where $\omega$ is positive. As ${\gamma }_{-}$ is slightly larger than $|{\gamma }_{+}|$ ^23^, and the difference between ${B}_{0}$, ${\omega }_{0-}/{\gamma }_{-}$ and ${\omega }_{0+}/|{\gamma }_{+}|$ is tiny, where ${\omega }_{0-}$ and ${\omega }_{0+}$ are respectively extracted from the resonance frequencies of the magnetic resonance spectrums excited by **B**_**c**_ and **B**_**cc**_, the resonance spectrum excited by **B**_**c**_ is for the lower ground-state hyperfine splitting, and the resonance spectrum excited by **B**_**cc**_ is for the upper ground-state hyperfine splitting. Based on equation (4) and the experimental conditions, we can deduce $\langle {F}_{-x\text{'}}\rangle$ and $\langle {F}_{-y\text{'}}\rangle$ excited by **B**_**c**_, which are approximately 0 and $0.008\hslash$ at ${\omega }_{0-}$, respectively, and $\langle {F}_{+x\text{'}}\rangle$ and $\langle {F}_{+y\text{'}}\rangle$ excited by **B**_**cc**_, which are approximately 0 and $0.037\hslash$ at ${\omega }_{0+}$, respectively.

As shown in Fig. 4, a linearly polarized oscillating magnetic field will excite magnetic dipole transitions of ^133^Cs atoms in both ground-state hyperfine levels. However, we can almost just excite ^133^Cs atoms in the lower (upper) ground-state hyperfine level using a clockwise (counterclockwise) rotating magnetic field, though ^133^Cs atoms in the upper (lower) ground-state hyperfine level are slightly excited due to the limit of the experimental condition that the applied clockwise (counterclockwise) rotating magnetic field is mixed with a minor counterclockwise (clockwise) rotating component.

**Figure 5:**
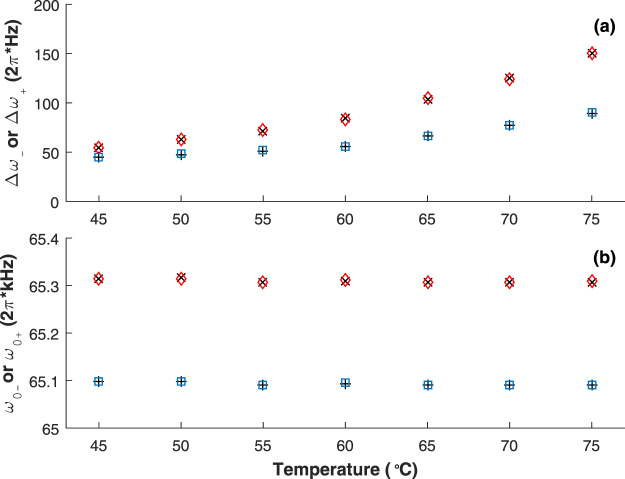
Linewidths (**a**) and resonance frequencies (**b**) of magnetic resonance spectrums for the lower and upper hyperfine splittings under different operational temperatures and exciting magnetic fields. The red diamonds are the experimental results for the lower hyperfine splitting when the exciting magnetic field is **B**_**c**_, the blue squares are the experimental results for the upper hyperfine splitting when the exciting magnetic field is **B**_**cc**_, the black crosses are the experimental results for the lower hyperfine splitting when the exciting magnetic field is **B**_**c**_ **+ B**_**cc**_, and the black pluses are the experimental results for the upper hyperfine splitting when the exciting magnetic field is **B**_**c**_ **+ B**_**cc**_.

In addition, as shown in Fig. 4, the magnetic resonance spectrum excited by ${{\bf{B}}}_{{\bf{c}}}+{{\bf{B}}}_{{\bf{c}}{\bf{c}}}$ is just a supposition of that excited by **B**_**c**_ and that excited by **B**_**cc**_. For further verifying this conclusion, the linewidths ${\rm{\Delta }}{\omega }_{-}$ and ${\rm{\Delta }}{\omega }_{+}$ and resonance frequencies ${\omega }_{0-}$ and ${\omega }_{0+}$ of magnetic resonance spectrums respectively for the lower and the upper hyperfine splittings are measured under different operational temperatures and exciting magnetic fields. By fitting the magnetic resonance spectrums excited by **B**_**c**_, **B**_**cc**_, and ${{\bf{B}}}_{{\bf{c}}}+{{\bf{B}}}_{{\bf{c}}{\bf{c}}}$, we can obtain corresponding ${\rm{\Delta }}{\omega }_{-}$ and ${\omega }_{0-}$, ${\rm{\Delta }}{\omega }_{+}$ and ${\omega }_{0+}$, and ${\rm{\Delta }}{\omega }_{-}$, ${\omega }_{0-}$, ${\rm{\Delta }}{\omega }_{+}$ and ${\omega }_{0+}$, respectively^23^, and the results are respectively shown with the red diamonds, the blue squares, and the black crosses and pluses in Fig. 5. As shown in Fig. 5, considering the measuring and fitting errors and the fluctuation of **B**_**0**_, the linewidths and resonance frequencies of magnetic resonance spectrums for the lower (upper) hyperfine splitting excited by **B**_**c**_ (**B**_**cc**_) and **B**_**c** +_ **B**_**cc**_ are almost identical, demonstrating the above conclusion.

The technique to selectively excite spin-polarized alkali atoms in different ground-state hyperfine levels has extensive application value, which is useful especially when ${\rm{\Delta }}{\omega }_{-}+{\rm{\Delta }}{\omega }_{+}$ is larger than ${\omega }_{0-}-{\omega }_{0+}$, or the difference of spin polarizations for alkali atoms in the two ground-state hyperfine levels is quite large. For example, it can be used to accurately measure spin relaxation and polarization of alkali atoms in one of the two ground-state hyperfine levels by avoiding the interference of alkali atoms in another ground-state hyperfine level, which is helpful for the research of optical pumping and relaxation mechanisms. In addition, this technique can also be applied into the Mx magnetometer to improve the measuring precision of external magnetic fields by avoiding the shift of the zero-crossing frequency of in-phase signal from the resonance frequency.

**Figure 6:**
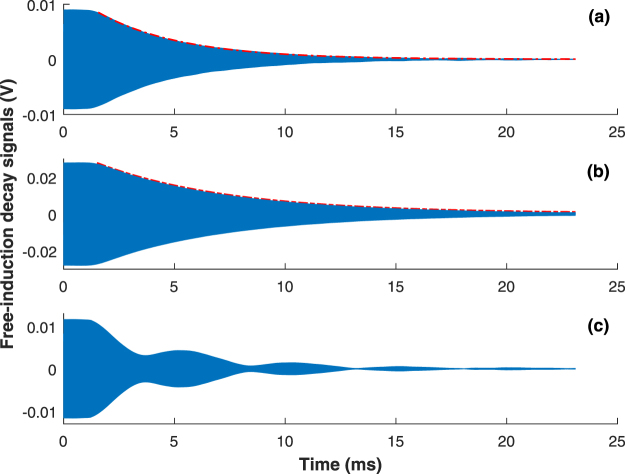
Free-induction decay signals under different exciting magnetic fields: (**a**) a clockwise rotating magnetic field, (**b**) a counterclockwise rotating magnetic field, and (**c**) a linearly polarized oscillating magnetic field.

Figure 6 shows an application of this technique, which is a measurement of the transverse relaxation time of ^133^Cs atoms in either ground-state hyperfine level using the conventional free-induction decay method. An exciting magnetic field with the resonance frequency is applied for a period of time and then turned off suddenly. The NI DAQ acquires the free-induction decay signal detected by the balanced photodetector. The blue solid lines in Fig. 6(a),(b), and (c) are the results when the exciting magnetic field is ${{\bf{B}}}_{{\bf{c}}}={B}_{1}\,\cos ({\omega }_{0-}t)\hat{{\bf{x}}}-{B}_{1}\,\sin ({\omega }_{0-}t)\hat{{\bf{y}}}$, ${{\bf{B}}}_{{\bf{c}}{\bf{c}}}={B}_{1}\,\cos ({\omega }_{0+}t)\hat{{\bf{x}}}+{B}_{1}\,\sin ({\omega }_{0+}t)\hat{{\bf{y}}}$, and $2{B}_{1}\,\cos ({\omega }_{0-}t)\hat{{\bf{x}}}$, respectively. As shown in Fig. 6(c), though the frequency of the exciting magnetic field is the resonance frequency for the lower ground-state hyperfine splitting, the transverse spin component of ^133^Cs atoms in the upper ground-state hyperfine level are created to a great degree, causing the transverse relaxation time of ^133^Cs atoms in the lower ground-state hyperfine level hard to be determined. However, as shown in Fig. 6(a) and (b), by selectively exciting spin-polarized alkali atoms in one of the two ground-state hyperfine levels, we can avoid the interference of alkali atoms in another ground-state hyperfine level. The red dash lines in Fig. 6(a) and (b) are the exponential fitting results. Based on the exponential fitting functions, we can easily obtain the transverse relaxation times of ^133^Cs atoms in the lower and the upper ground-state hyperfine levels, which are 3.82 ms and 5.93 ms, respectively.

## Conclusions

In summary, we have theoretically and experimentally demonstrated a technique to selectively excite spin-polarized alkali atoms in different ground-state hyperfine levels by controlling the direction of an applied rotating magnetic field, which can separately create the transverse spin component of spin-polarized alkali atoms in either ground-state hyperfine level. This technique has extensive application value. It can be used to accurately measure spin relaxation and polarization of alkali atoms in either ground-state hyperfine level, which is helpful for the research of optical pumping and relaxation mechanisms. In addition, it can also be applied into the M_x_ magnetometer to improve the measuring precision of external magnetic fields.
